# Live Genomics for Pathogen Monitoring in Public Health

**DOI:** 10.3390/pathogens3010093

**Published:** 2014-01-21

**Authors:** Giuseppe D’Auria, Maria Victoria Schneider, Andrés Moya

**Affiliations:** 1 Área de Genómica y Salud, Fundación para el Fomento de la Investigación Sanitaria y Biomédica de la Comunidad Valenciana (FISABIO-Salud Pública), Avenida de Cataluña 21, 46020 Valencia, Spain; E-Mail: andres.moya@uv.es; 2 CIBER en Epidemiología y Salud Pública (CIBEResp), C/ Melchor Fernandez Almagro 3-5, Madrid, Spain; 3 The Genome Analysis Centre, Norwich Research Park, Norwich, UK; E-Mail: vicky.sg@tgac.ac.uk; 4 Instituto Cavanilles de Biodiversidad y Biología Evolutiva, Universitat de València, C / Catedrático José Beltrán 2, 46980 Paterna-Valencia, Spain

**Keywords:** pathogens outbreaks, pan-genome, comparative genomics, bioinformatics, resistance, public health

## Abstract

Whole genome analysis based on next generation sequencing (NGS) now represents an affordable framework in public health systems. Robust analytical pipelines of genomic data provides in a short lapse of time (hours) information about taxonomy, comparative genomics (pan-genome) and single polymorphisms profiles. Pathogenic organisms of interest can be tracked at the genomic level, allowing monitoring at one-time several variables including: epidemiology, pathogenicity, resistance to antibiotics, virulence, persistence factors, mobile elements and adaptation features. Such information can be obtained not only at large spectra, but also at the “local” level, such as in the event of a recurrent or emergency outbreak. This paper reviews the state of the art in infection diagnostics in the context of modern NGS methodologies. We describe how actuation protocols in a public health environment will benefit from a “streaming approach” (pipeline). Such pipeline would include NGS data quality assessment, data mining for comparative analysis, searching differential genetic features, such as virulence, resistance persistence factors and mutation profiles (SNPs and InDels) and formatted “comprehensible” results. Such analytical protocols will enable a quick response to the needs of locally circumscribed outbreaks, providing information on the causes of resistance and genetic tracking elements for rapid detection, and monitoring actuations for present and future occurrences.

## 1. Introduction

### 1.1. Following Microbes in Public Health Microbiology

Care units such as oncology and surgery, where patients are in most cases under conditions of immunodepression, are known for the presence of “the usual suspects” such as multi resistant *Pseudomonas aeruginosa*, *Escherichia coli* ESBL and *Staphylococcus aureus* MRSA. These are often the last obstacle to the clinical evolution of the patients. Around 0.1% of patients suffer sepsis every year whereas 20%–40% of these die in hospital. Without a deep knowledge of the organisms causing the sepsis, empirical antibiotic treatment is the first practice applied to stop the infection [1]. Guidelines for empirical therapy have to take into account the epidemiology of microbes isolated in care units. Intensive care units and hospitals are major reservoirs for pathogenic opportunistic organisms. Succeeding in eradicating the infection is mainly a race against time coupled with the selection of the proper empirical antibiotic treatment and the capabilities of bacteria in exchanging or evolving a variety of factors such as resistance, virulence and persistence [2]. Although the wide use of antibiotics contributed to the eradication of many diseases, continuous changes in trends of antibiotic resistance are observed [3]. With the advent of NGS the challenge is now to provide the public health sector with tools for fast and robust characterisation of pathogenic organisms, particularly for those cases in which difficulties in eradication emerge.

Resistant bacteria can emerge by a selective process in a particular population, fixing mutation conferring antibiotic resistance or by colonisation or infection with drug-resistant organisms already present in the surrounding environment [4]. Antibiotic susceptibility is routinely tested in *in vitro* assays after obtaining the isolates. The common accepted method to test antibiotic susceptibility is still officially based on minimum inhibitory concentration. Break points for antimicrobial susceptibility are periodically revised by specific organisms, for more information see “The European Committee on Antimicrobial Susceptibility Testing” [5]. In intensive care units, the first data about bacteria susceptibility are provided within 48 h. This time frame allows antibiotic treatment adjustments to eventually be made. Several cases showed that the emergence of antibiotic resistant bacteria leads to ineffective treatments [6,7]. Rapid antibiotic resistance profiles are thought to highly improve the quality of therapies, reducing, on the other hand, side effects such as commensal over-infections or the generation of new resistant strains [8]. Exceptions where cohort based studies do not show a significant association between the application of the appropriate empiric antimicrobial therapy and in-hospital post-infection length of stay or mortality have also been observed [9,10]. However, when eradication of a given infection is delayed or when an outbreak is extended, a more detailed gathering of information can help in resolving the emergency.

### 1.2. Comparative Genomics in Public Health

Modern public health microbiology laboratories have means to isolate strains of interest, since most of the micro-organisms under surveillance can be accounted for with standardised isolation methodologies. At present, it is possible to store, organize and maintain the organism’s genetic/genomic data and its associated metadata in bio-banks [11]. Historically, public health microbiology units base their daily work on classical microbiology practices. Several metabolite based kits and gene PCR-based systems allow identification with good approximation of the presence of specific and/or most common groups of bacteria. Current trends show how some of these extend to also recognising specific resistance factors [12,13,14]. These kits can detect the presence of the bacteria or the antibiotic resistance factor directly from blood avoiding the time lapse for cultivation. The limitation lies in the lack of continuity in updating the recognition power in terms of new organisms or resistance factors appearing, not only in terms of time, but also in terms of geographic spaces. Ideally, a new outbreak or a failure in antibiotic treatment is due to a change in microbial resistance or persistence profiles. This should be considered as a temporal and local (geographical) factor. Commonly observed is the appearance of dangerous nosocomial outbreaks which are geographically or temporally defined [15,16,17]. For these, deeper studies based on the whole genome sequencing causing the outbreak will be suitable for gaining insights into the infection and resistance mechanism adopted by the pathogen. Whole genome based monitoring during outbreaks as in the case of *Legionella pneumophila*, *Mycobacterium tuberculosis*, *E. coli*, *etc.* point out the importance of working with whole genome data in a comparative framework, highlighting taxonomy relationships, mutation based clustering, orthologues and accessory genes distribution. The latter is considered to be a main source for resistance, virulence and persistence factors [18,19,20,21].

Nowadays, whole genome sequencing protocols are relatively easy to apply in order to solve daily problems. Probably, the most pragmatic approach relies on the proposal of surveillance units of candidate organisms to be sequenced. When this actuation plan starts, the same microbiology unit can proceed through DNA extraction of the target organism and easily pass the DNA sample(s) to a specialised service for sequencing through the most appropriate NGS methods. We have to keep in mind that an alert from a surveillance unit can justify the expense for a whole genome sequencing project, the costs for which are predicted to be continuously reduced due to the further development of sequencing technologies. When the sequencing unit returns the obtained sequences, these can flow through pipelines for data mining and extraction of the information required by surveillance and microbiology units for further decisions and actuations.

In this paper, we describe a “live” frame-work in microbial genome sequencing in which data mining from comparative genomics continuously populate a relational database with genetic information, allowing the extraction of useful differential data of interest such as virulence, resistance persistence factors, SNPs and InDels. Such approaches make data consequently suitable for immediate designing of new diagnostics systems for early predictions of organisms bringing potential dangerous features. Future advances in public health microbiology would entail services based on provision of large scale comparative analysis of organisms belonging to the same species.

For instance, the Global Microbial Identifier (GMI) initiative has started work in this direction promoting whole genome sequencing of organisms with public health relevance. GMI aims to store data, compare genomes and identify genes which could be of interest in outbreak characterisation as well as describing emerging pathogens [22].

Theoretical and technological advances are ready to support all processes from strain collection, DNA purification and storing. We are at the turn of microbiology and genomics research towards providing multiple genomic information from most similar organisms, analysing commonly shared features (or core genome) and additional characters (or disposable genome), in other words, we are in the era of the “pan-genome”.

### 1.3. Approaching Species Definition in the Genomics Era

One of the most discussed issues in microbiology is the species definition in taxonomy. Since the early 1970s, molecular methods and DNA sequencing techniques have been adopted to provide objective criteria in defining bacterial species. At first, whole genome DNA-DNA hybridisation was used to determine when two strains were homologous. Johnson (1973) [23] determined that strains from the same species nearly always shared 70% or more of their genomes. However, variation in gene content and the presence of polymorphisms among strains assigned by DNA-DNA hybridization to the same species have led some to consider that such a “species concept” is far too broad, compared to those in organisms of higher complexity than bacteria [24]. Other methods have been proposed to define microbial species based on diverse cut-off values studying one or more genes. 16S taxonomy, Multi Locus Sequence Typing (MLS or MLST) and biochemical characterisations are some of the most accepted classification methods [25,26]. Whereas none of these methods describe the complete genetic repertoire, whole genome analysis represents the gold standard in comparative genomics always permitting *a-posteriori* single or multiple gene-based characterisations [27]. Pathogenic organisms of interest could be tracked at the genomic level, monitoring at the same time its expansion, pathogenicity, resistance to antibiotics, virulence and persistence factors, mobile elements, adaptation features, all in a geographic context. Though several reference genomes are available, it is worth memtioning the efforts needed for sequencing and assembling *de-novo* without a reference backbone [28,29]. The American Center for Disease Control and Prevention (CDC) promotes whole genome sequencing for real time epidemiology, where results are expected to provide prospective information about outbreak evolution. The European Centre for Disease Prevention and Control (ECDC) is working in this direction, focusing on *how public health can benefit from the rapidly evolving NGS technology in molecular microbiology* [30]. While more and more reference genomes are available, comparative analysis represents a must in public health microbiology, offering the possibility to discover differential traits involved in the pathogenicity of a given organism.

## 2. Pan-Genome

**Figure 1:**
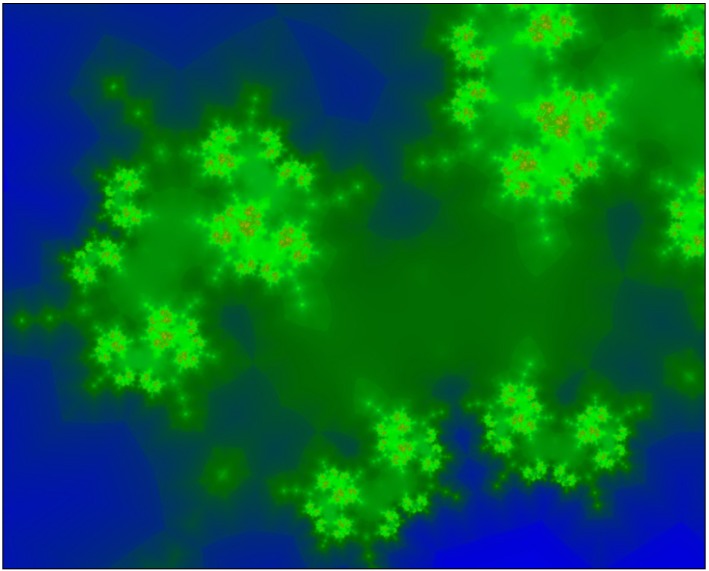
Fractal evolution model. Artwork describing ideally a fractal evolution model showing the outcome of new offspring (light green islands) with fitness advantages which are fixed and explode (darker islands nuclei), although the evolutionary pattern is maintained (fractal periodicity). On the other hand, small generations could be slower in their evolution or disappear in the time lapse. This model is established by representing a continuum among all organisms inhabiting a given environment.

Tracking and comparing genomes entails studying of the genomic difference among compared strains. The term “pan-genome” refers to *pan* (from Greek παν, whole) and *genome* (genome) referring to the inclusion of the core and the dispensable genome [31,32]*.* While originally microbial expansion was thought as clonal, now it is well known that also the overnight culture bacteria go through substitutions, insertions and deletions due to polymerases errors, genomic rearrangements (e.g. mobile elements) and viral interactions. Bacteria isolated from the same environment can show important genomic differences due to the accumulation of variations during their natural life cycles [33]. The differences in accumulation of variations are attributed to the different speeds and scales for all organisms in a given environment as a “*fractal pattern*” in evolution (Figure 1).

This continuous evolution process, jointly with the genetic enrichment provided by horizontal gene transfer events, prevent the genome of a bacteria isolated from a given environment overlapping with other genomes of other organisms defined as being taxonomically the same (in terms of 16S rDNA, MLST or DNA/DNA hybridisation profiles), which may have been isolated at different times or from different locations [34,35]. Central metabolic genes are mostly found as orthologues in all genomes of bacteria belonging to the same taxa, (common genome, shared genome or, most used, core-genome). Additional genes (dispensable genome) seem to be the ones making the difference [36]. For instance, antibiotic resistance factors, toxin/anti-toxin systems or phage-resistance clusters are considered in bacteria as arsenals for maintenance, evolution and transferring, making them a challenge in public health due to how difficult they are to track and control [37,38,39,40].

### 2.1. Core Genome

By analysing bacteria belonging to the same species at a whole genome level, comprehensive comparative genomics can be carried out. Shared genes among multiple strains are mostly related to house-keeping genes or central metabolic processes, most of the structural information and main genotypic features. The core genome could be thought as the number of shared features in a pool of genomes. The size of the core genome decreases, increasing the number of genes added to the pool. While it is possible to link the core genome to common tracts among considered bacteria, it is worth mentioning that such a calculation also depends technically on the number of genomes available for the computation. In this review, we propose a comparative analysis example of some organisms considering all genomes currently deposited in GenBank of *Campylobacter jejuni* (nine genomes), *Streptococcus suis* (13 genomes), *L. pneumophila* (10 strains), and *Staphylococcus aureus* (31 genomes, see SI1 for a strain list).

### 2.2. Dispensable-Genome

As long as new genes (by definition as part of dispensable compartment of the pan-genome) are added in the computation to the pool, the volume of the pan-genome increases. The increase of the pan-genome size has been observed to be either faster or slower. This has created another concept of the open or closed pan-genome [41]. A species pan-genome is considered closed, when as many new genomes are added to the pool and no new genetic information appears. For example, *Streptococcus agalactiae* pan-genome can exceed at least three-fold the average genome size [32]. On the other hand, highly adapted bacteria, especially those characterised by living in very restricted environmental niches, such as host specific pathogens such as *Salmonella paratyphi* or *Bacillus anthracis,* show a closed pan-genome [36,42].

The dispensable genome, also defined as “accessory” or “adaptive genome” [32], includes genes conferring adaptive advantages to the strain in order to survive in a specific environment. In most cases, these factors are linked to antibiotic resistance, virulence, capsular serotype, adaptation, and might reflect the organisms predominant lifestyle [40,41]. Being aware of differential traits in terms of presence/absence of genes and their annotations means having knowledge about the versatility or pathogenicity of a given organism separate from the pure taxonomic position. A recent study by den Bakker and collaborators (2011) offers a good example of comparative genomics applied to the identification of evolutionary clades of *S. enterica* subsp. *enterica.* Here, the authors provide a population genetic framework for studying the virulence and propagation of this pathogen. In their work, 46 complete genomes of *S. enterica*, 16 new genomes sequenced using SOLiD^TM^ system and 30 genomes already present in GenBank were analysed. *S. enterica*’s pan-genome was calculated, and common and different genomic traits were spotted by identifying in the two clades what differed in terms of metabolic capabilities, adhesion and colonization properties. Studying two clades of *S. enterica* subsp. en*terica* at the level of its pan-genome highlighted the existence of conserved pathogenicity islands and a virulence gene repertoire [43].

In 2010, D’Auria *et al.* described the pan-genome of *L. pneumophila* (five genomes at that time) revealing strain-specific and common traits including anti-drug resistance systems; a system for transport and secretion of heavy metals; three systems related to DNA transfer; two CRISPR systems, known to provide resistance against phage infections; and seven islands of phage-related proteins, five of which seem to be strain-specific and two shared among compared genomes [40].

## 3. SNPs/InDels Profiles

Whole genome sequencing allows having at one time the whole SNPs and InDels profiles for each gene in a multi-genome context [44]*.* Whole genome taxonomy was reviewed in depth by Rannala and Yang in 2008 [45]. Phenotype identification and genotypic typing techniques were mentioned as the basis for infectious disease epidemiology, providing profiles that are of use, not only for taxonomic reconstruction, but also for tracking strains during outbreaks [46]. The *Yersinia pestis* plague, characterised by a genetic uniformity, made it easy to be traced at the global level. Multi-genome SNPs profiles allowed to define its origin in or around China migrating from East to West. SNPs lineages were then traced highlighting the radiation to Europe, Africa and South-east Asia, while North American radiation originated from a single point [47]. The availability of such fine tools for lineage tracking is of great interest. In a public health frame-work, it is probably not useful, nor possible, to sequence all genomes of organisms from an outbreak episode, but having the genome of some representative strain would help towards obtaining a deeper knowledge about the factors responsible for resistance complicating infection eradication. While data mining processes allow the identification of statistically relevant SNPs/InDels, applied science will provide the basis to develop new tracking PCR-based systems. PCR detection systems have been successfully applied with several genes of interest and are often included in commercial test kits [48,49,50]. In this context, a “live” or continuously growing SNPs/InDels database would highly contribute to the prediction of polymorphisms suitable for test kits development, not only with globally applicable purposes, but even more interestingly, at small local scale (hospitals, villages, city, *etc.*). In other words, having polymorphisms’ profiles for bacteria of interest belonging to a specific outbreak would help to elaborate a short time test for its immediate identification and tracking.

## 4. Automatic Pipelines

**Figure 2:**
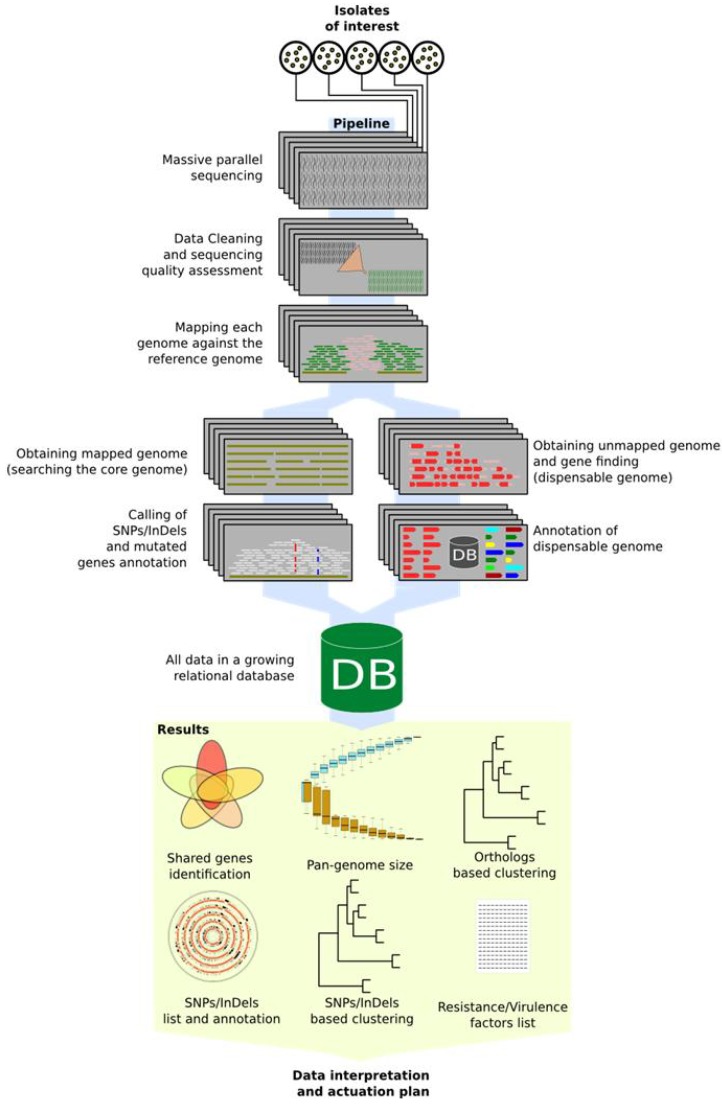
Automatic data mining pipeline schema. Picture shows proposed schema from strains selection to data reading for actuation plans. All steps over blue path are automatic and do not need user supervision.

While it seems impossible to afford the sequencing and perform the bioinformatics tasks in a routine daily work time frame for a microbiology unit in public health, automatic pipelines represent the solution. These will help automatising several of the necessary steps and tasks providing the user with the final data required in a round time compatible with the work beat. While the number of software for NGS data analysis is continuously growing [51,52,53], existing pipelines can be integrated and extended according to the microbiology unit needs. In terms of reducing human intervention, pipelines can be designed to cover as much of the analytical tasks as possible, not only for quality assessment and ancillary data production, but also for data mining and visualisation. Figure 2 reports a schematic pipeline for NGS data production and data mining, starting from sequencing of organisms of interest to gathering of useful data.

Among the data which a microbiology unit needs to know in a “live” context, we also suggest including properties linked to genes or to specific mutations, in other words, the pan-genome relationship of a given isolate within its species and the mutational (SNPs/InDels) profile. This kind of automatic pipeline provides the user, in a short frame of time, with differential genes data (dispensable genome) as well as with mutation profiles which allow positioning of the studied strain in a phylogenetic context.

In our example, an automatic pipeline was applied to simulated NGS data obtained using Illumina error profiles [54] on complete genomes of *Campylobacter jejuni*, *Streptococcus suis*, *L. pneumophila* and *S. aureus* strains (Table 1 and Table SI1).

The applied pipeline starts from sequence data going step-by-step from data cleaning and quality assessment through the production of useful intermediate data characterising, on one hand gene-related data in a pan-genomic context, and on the other hand, the mutation profiles.

**Table 1:** Used genomes and main genomic features. SI1 reports accession numbers for each genome of each species. Orthologues’ assignation to define pan-genome size was carried out by clustering all genes of each organism dataset with at least 60% similarity (amino acid) and 60% sequences overlapping using CD-HIT program [55].

	*C. jejuni*	*S. suis*	*L. pneumophila*	*S. aureus*
Number of genomes	9	13	10	31
Average genome length	1,678,553.8 bp	2,090,478.5 bp	3,302,389.7 bp	2,894,586.7 bp
Pan-genome size	953	1,125	1,933	1,765

### 4.1. Pipeline—A Little Bioinformatics

Pipelines are thought to reduce human intervention, maintaining analysis as robust and reproducible. Almost all of the necessary steps can be run consecutively one after the other, feeding the next step with the output of the previous one. The applied pipeline goes through several concatenated steps through the quality assessment process using “PRINSEQ” program [56], mapping using “SMALT” program [57], consensus definition and data conversions by the use of SAMtools [58], *de-novo* assembly of unmapped reads by MIRA program [59], gene finding using GLIMMER3 [60], automatic annotation using Hidden Markov Models algorithm searching in PFAM database [61,62,63], SNPs/IndDels calling by the use of VarScan program [64], and almost all required statistics have been performed in R environment [65,66]. The actual data report can be obtained using appropriate software which allows script execution and which could be concatenated with data-mining steps.

### 4.2. Pan-genome Data Mining Pipeline

**Figure 3:**
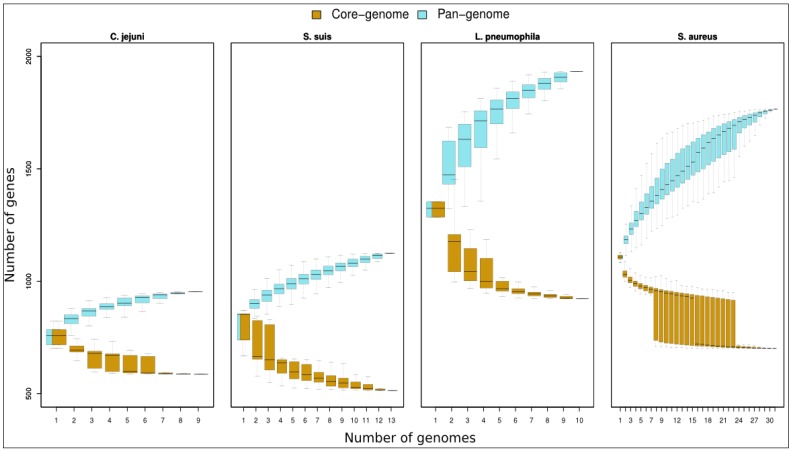
Core genome distributions. Graph shows boxplots of pan-genome and core genome contents for increasing pools of genomes belonging to *C. jejuni*, *S. suis*, *L. pneumophila* and *S. aureus*. In *S. aureus* graph, we considered 31 genomes; due to the elevated number of possible combinations of genomes pools from n = 8 to n = 23 the boxes describe sampling of 2000 random combinations.

The interest in pan-genomic data relies on discovering additional features of a given organism. In this context, a closed or open pan-genome can provide an idea about the versatility of the studied strain. Generally, we face organisms with a reduced ecological niche such as *C. jejuni* (Figure 3, left panels), where the increase in pan-genome size slows down within the nine genomes considered (almost closed pan-genome) or with organisms such as *L. pneumophila* or *S. aureus* (Figure 3, right panels), inclined to horizontal gene transfer and characterised by having an open pan-genome.

**Figure 4:**
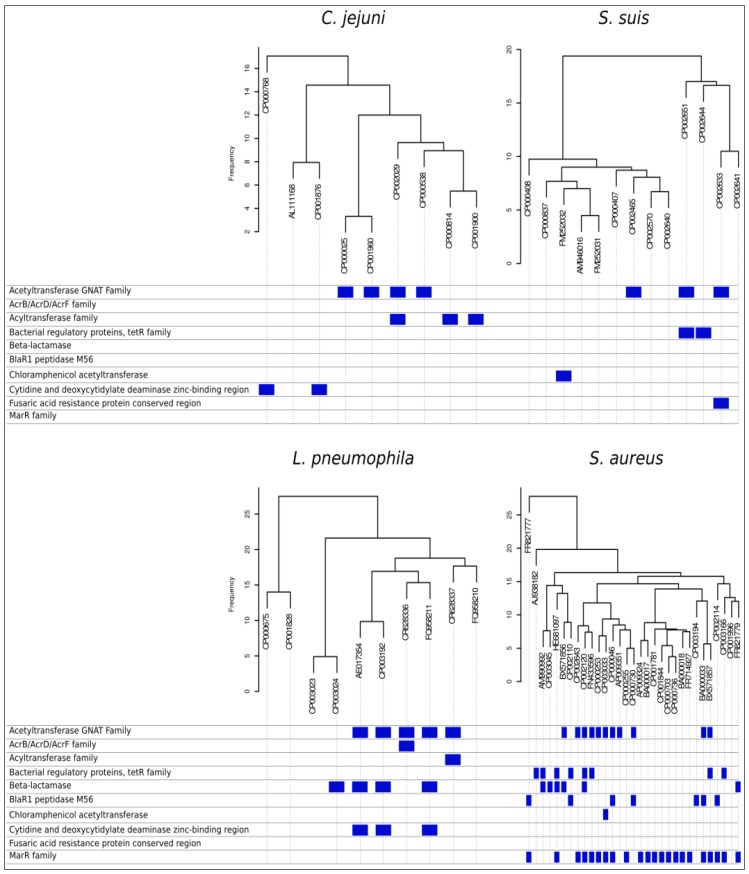
Antibiotic resistance factors. Dendrograms shows distribution among strains of *C. jejuni*, *S. suis*, *L. pneumophila* and *S. aureus* based on presence/absence of orthologues genes. For each genome, the presence of dispensable genes related with antibiotic resistance is identified, and by applying automatic pan-genome analysis, the pipeline is reported.

Further, orthologue distributions provide the user with gene profiles for each organism, identifying differential tracts among organisms. When the mapping step of the pipeline runs by identifying reads overlapping with reference genome, unmapped reads can be tracked and associated to parts of the genome not present in the reference genome. These reads can be *de-novo* assembled and annotated, contributing towards revealing what makes the strain special with respect to previous known genomes. Annotating differential genes highlights the presence of pathogenicity factors. Clustering organisms by their differences in genes presence/absence and frequency allows stratifying annotation information, in terms of differences in versatility and pathogenicity. Figure 4 shows orthologues based dendrograms for the four species used as examples. Below each dendrogram, blue marks indicate some of the differential gene features related to antibiotics resistance factors highlighted by the data mining pipeline. For instance, several strains among all genomes reported resistance factors to betalactamases with different mechanisms that only a fast comparative analysis can bring to light. SI2 reports the complete table of differential genes encountered among all considered strains of the four pan-genomes.

## 5. Conclusions

NGS coupled with automatic data mining pipelines represent nowadays the future for promptly definition of actuation plans responding to outbreaks or recurrent infections in public health systems. This framework is even more useful in small scale situations where a specific and proper action is needed. The speed and robustness of NGS methodologies and strategies now make possible the production of genetic and mutation profiles within a couple of days, making the automatic data mining process compatible with the immediate need of information in emergency cases. In the case of genetic data, differential gene profiles obtained comparing the strain(s) of interest with the ones already present in databases allows the definition of its pan-genome, thus data mining pipelines can reveal to the users which genes are making the difference in terms of antibiotic resistance or environmental persistence factors. Moreover, mutation profiles provide users with the correct information for taxonomic identification of the proposed strain on a clonal scale as well as with possible targets for the fast development of tracking systems kits, based, for example, on PCR methods. An important technical challenge is surely represented by the data storage and data mining process in such a growing frame-work. The experience from information and communication technology will probably be of help in optimising automatic pipeline processes and data flows avoiding redundancies and focussing the attention mainly on differential tracts labelling and flagging. Finally, having these data in relational databases interconnected among multiple centres of public health system in the future will be key to creating the “live” context for modern genome-based microbiology.

## Supplementary Files

Supplementary File 1 Supplementary Information (ZIP, 41 KB)
